# Policy interventions to improve the accessibility and affordability of Dutch dental care. A scoping review of effective interventions

**DOI:** 10.1016/j.heliyon.2024.e28886

**Published:** 2024-04-17

**Authors:** E.H. Wolf, K.A. Ziesemer, E.J.E. Van der Hijden

**Affiliations:** a Talma Instituut, Vrije Universiteit Amsterdam, Faculty of Social Sciences, De Boelelaan 1105, 1081, HV Amsterdam, Noord-Holland, the Netherlands; b Amsterdam UMC, Vrije Universiteit Amsterdam, Medical Library, De Boelelaan 1117, 1081, HV Amsterdam, Noord-Holland, the Netherlands; c Zilveren Kruis Health Insurance, Handelsweg 2, 3707 NH Zeist, Utrecht, the Netherlands

## Abstract

Caries and periodontitis remain prevalent in the Netherlands. Given the assumption that increasing the accessibility and affordability of dental care can improve oral health outcomes, policy interventions aimed at improving these aspects may contribute to better oral health. To identify possible solutions, this scoping review firstly identifies policy interventions from around the world that have effectively improved the accessibility or affordability of dental care. Secondly, this review discusses the potential of the policy interventions identified that are applicable to the Dutch healthcare sector specifically.

A literature search was performed in four databases. Two reviewers independently screened all potentially relevant titles and abstracts before doing the same for the full texts. Only studies that had quantitatively evaluated the effectiveness of policy interventions aimed at improving the accessibility or affordability of dental care were included. 61 of the 1288 retrieved studies were included.

Interventions were grouped into four categories. Capacity interventions (n = 5) mainly focused on task delegation. Coverage interventions (n = 25) involved the expansion of covered dental treatments or the group eligible for coverage. Managed care interventions (n = 20) were frequently implemented in school or community settings. Payment model interventions (n = 11) focused on dental reimbursement rates or capitation. 199 indicators were identified throughout the 61 included studies. Indicators were grouped into three categories: accessibility (n = 137), affordability (n = 21), and oral health status (n = 41).

Based on the included studies, increasing managed care interventions for children and adding dental coverage to the basic health insurance plan for adults could improve access to dental care in the Netherlands. Due to possible spillover effects, it is advisable to investigate a combination of these policy interventions. Further research will be necessary for the development of effective policy interventions in practice.

## 1 Background

Although largely preventable, oral diseases such as periodontitis and caries are among the most prevalent diseases globally [1]. In the Netherlands, oral health is maintained at a relatively high standard, but periodontitis and caries remain prevalent. Almost 40% of Dutch adults had an unhealthy periodontium in 2013, and in 2017 80% of the 23-year-olds had at least one decayed, missing or filled tooth [[2], [3], [4]]. Oral health status is affected by factors such as age, overall health, and migration background [4,5]. Socioeconomic status is also correlated with oral health, with the most deprived groups being most often affected by caries and periodontitis [6]. These disparities are reflected in dental visits: while 80% of the Dutch population visits a dentist at least once a year, about 1.5 million adults do not regularly visit a dentist due to financial reasons [7,8]. In recent years, the percentage of 4- to 7-year-old children who have yet to visit the dentist was 15%. Among children even younger than 4 years old, this percentage has reached as high as 60% [9]. Disparities also exist in the dental care that is delivered. For example, removable prosthetics are more common among groups with a low socioeconomic status, whereas fixed prosthetics are regularly used within groups with a higher socioeconomic status [6]. Such an example reveals not only that there is still room for improvement in the oral health status of the Dutch population, but also that disparities in the accessibility and affordability of dental care still exist. At this moment, it is unclear which policy interventions could potentially help overcome these problems.

*Research context: the organization of dental care within the Netherlands* Up until 1995, dental care for the lowest income groups was publicly covered by national health insurance. In 1995, dental care was excluded from this public coverage and replaced by the possibility of purchasing private, supplementary dental insurance [10]. Then in 2006, when the Health Insurance Act took force, all residents became obliged to sign up for basic health insurance through a private health insurer. Since then, adults can apply for supplementary dental insurance – which, in 2021 for example, 79% of Dutch adults opted to do [11]. Dental coverage differs both per health insurer and per insurance package in terms of reimbursed treatment costs and height of copayments [11]. Only dental care for specified patient groups, care provided by an oral surgeon, and care regarding prosthetic facilities in the edentulous jaw are covered by the mandated basic health insurance package. Preventive and curative dental care for children up to 18 years is completely covered by every basic health insurance plan [12].

At present, dental care is delivered almost completely in private dental practices. At the beginning of the past century, public dental care was introduced at primary schools. Twice a year, a dental check-up would take place and, when needed, curative care would be delivered. At the end of the 1980's, governmental budget cuts and increasing competition between private dental practices led to the gradual disappearance of many school dentists. However, public pediatric dental care is still offered up to this day in a few of the Netherlands' biggest cities [13].

Dental care is currently financed through a fee-for-service payment model. Fees are fixed and defined on a yearly basis by the Dutch Healthcare Authority [14]. Although the possibility of negotiating lower prices technically exists, it is normal practice for dentists and dental hygienists to get reimbursed for the maximum price. About 85% of dentists currently work as a practice owner or as an independent contractor without their own practice. The remaining group of dentists is remunerated through an employment contract, many of whom are, for example, specialized dentists working in hospital settings to treat people with physical or mental disabilities [15,16]. In recent years, young dentists have increasingly chosen to work as independent contractors instead of owning their own practice. At the same time, more and more practices are being bought by dental chains. Nowadays, about 10% of all dental practices in the Netherlands are currently part of a dental chain [15,17].

10,200 dentists and 3900 dental hygienists are currently registered in the Netherlands [15]. These numbers appear too low to cover the whole demand for oral health care, especially in rural areas. This shortage of dentists is partly due to an absolute increase in dental care demand, as the share of people maintaining their own dentition as they age increases. However, other factors, like an uneven distribution of oral care providers across the country and feminization of the dental profession (due to Dutch women being more likely to work part-time), contribute to the shortage as well [[15], [16], [17]]. In 2020, a five-year experiment of redistributing tasks from the dentist to the dental hygienist was started to address shortages of oral care providers [16].

*Research aim* Notwithstanding that oral health status is greatly affected by people's own health behavior, we hypothesize that improving the accessibility and affordability of dental care will also improve the country's overall oral health status. Many countries are facing problems comparable to those of the Netherlands as they work to ensure sufficiently accessible and affordable dental care [18]. As such, policy interventions that have been implemented in other countries may prove interesting for the Netherlands as well. By conducting an international scoping review of the literature, this study gives an overview of these policy interventions while aiming to answer the following research question: which policy options have improved the accessibility and affordability of dental care around the world, and which of those can be applied within the Netherlands given the characteristics of Dutch dental care?

## 2 Methods

A systematic scoping review of the literature was conducted to give insight into the effectiveness of policy interventions aimed at improving the accessibility and affordability of dental care. Due to the broad nature of the research question, the complexity of the literature, and a lack of conceptual boundaries, a scoping review was considered an appropriate research method [19]. Both the conduct and reporting of this review adhere to the Preferred Reporting Items for Systematic Reviews and Meta-Analyses (PRISMA) Statement [20]. Likewise, this review was written in accordance with the guidance for scoping reviews issued by the Joanna Briggs Institute [21].

### 2.1 Search

After conducting several initial scoping searches, four bibliographic databases were searched for relevant literature from their inception up to June 17, 2022: PubMed, Clarivate Analytics/Web of Science Core Collection, Elsevier/SCOPUS, and ProQuest/International Bibliography of the Social Sciences. Each search was devised in collaboration with a medical information specialist (KAZ). Search terms — including synonyms, closely related words, and keywords — were used as index terms or free-text words, for example: “dental care,” “policy,” “payment,” and other statistics-related terms. The searches did not contain any methodological search filters, dates, or language restrictions that would have limited results to specific study designs, dates, or languages.

Duplicate articles were excluded using the R-package “ASYSD,” an automated deduplication tool [22], followed by manual deduplication in Endnote (X20.0.3) by the medical information specialist (KAZ). Appendix A details the full search strategy used for each database.

### 2.2 Selection process

**Fig. 1:**
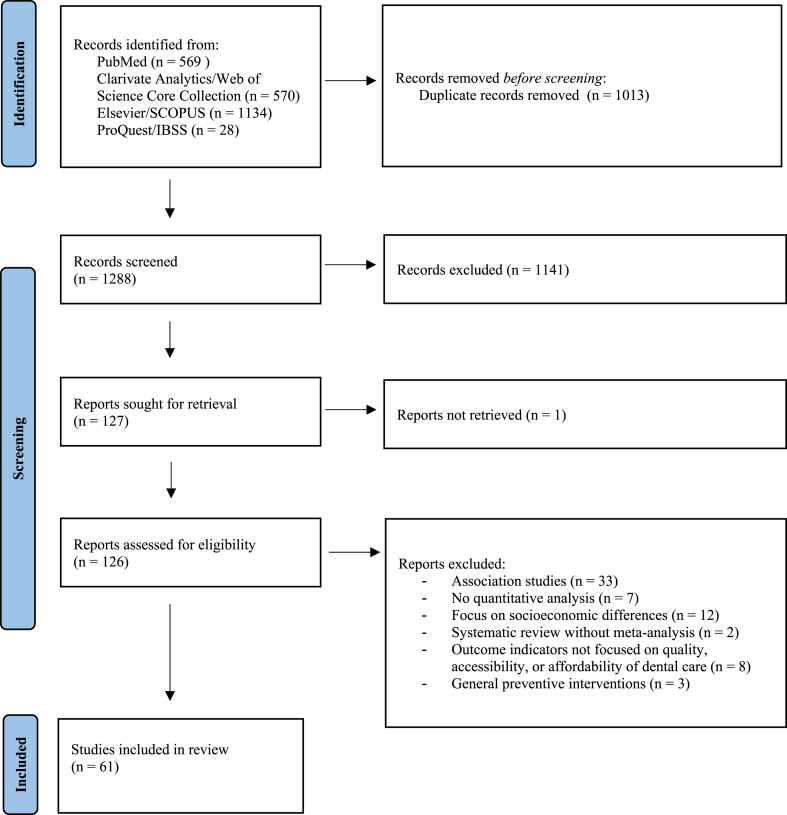
Flow diagram of screening process.

Two reviewers (EW and EVDH) independently screened all potentially relevant titles and abstracts for eligibility using Rayyan [23]. When necessary, the full article was checked for adherence to the eligibility criteria. Differences in judgement were resolved through a consensus procedure, which entailed that studies would be included if they met both of the following criteria: (i) the study empirically assessed the effectiveness of a dental policy intervention for the general population; and (ii) indicators of the study focused on the accessibility and/or affordability of dental care. We excluded any cost-effectiveness studies and clinical dental interventions in case they were not linked to a policy intervention. We also excluded studies that specifically focused on the reduction of socioeconomic differences in dental care, as these studies were too specific to fit our review. Generic policies like the fluoridation of tap water were also excluded, as these policies did not directly affect the organization of the dental sector. Editorials, letters, legal cases, and interviews were excluded as well. Systematic reviews in which meta-analysis had not been performed were also excluded, although the studies included in those reviews were added to the results and subjected to consideration via the selection process. Fig. 1 illustrates this screening process, including the applied exclusion criteria.

### 2.3 Data charting

First, each study was analyzed to identify its author, country of study, year of publication, intervention characteristics, and indicators. Focusing on the intervention characteristics, we were then able to extract four main policy categories: capacity, coverage, managed care, and payment models. Each study included in this review was put into one of these categories. Next, interventions were classified by their targeted population group: children, adults, older persons, pregnant women, or the population at large. Indicators were then combined into groups based on unifying dynamics, for example: “preventive dental care,” “oral examination,” “dental examination,” and “scaling usage” were combined into one main category named “preventive dental service.” Distinctions were consistently drawn between indicators assessing provided dental care and those measuring received dental care.

Next, all indicators were labeled according to their association with the intervention's goal: to increase either the accessibility or affordability of dental care. Indicators that were related to use of care, or the magnitude of the dental workforce, were categorized under the goal of accessibility. Indicators concerning (out-of-pocket) expenditures on oral health were categorized under the goal of affordability. In addition to these two intervention goals, we also set up a third indicator category for oral health status, as many studies also investigated the effects of an intervention on health outcomes; for example, “X percent of children with tooth decay” or “self-reported oral health.” Depending on the study and design of the intervention, some indicators were classified as being associated with more than one goal.

To determine whether the evaluated interventions in the studies had a positive or negative outcome, it was noted for each study whether a decrease or an increase in the outcome indicators was seen as positive and whether this effect was significant or not significant. By doing this, all studies were given one of the following labels: positive, negative, or no effect.

When a study reported different effect sizes for different target groups, the highest level of aggregation for the associated indicator was reported. When studies evaluated indicators over time, the most recent reported values were recorded.

## 3 Results

The literature search generated a total of 2301 references: 569 in PubMed, 570 in Clarivate Analytics/Web of Science Core Collection, 1134 in Elsevier/SCOPUS, and 28 in ProQuest/International Bibliography of the Social Sciences. After removing duplicates of the references that had been retrieved by more than one database, 1288 references remained. Of these references, 147 were found eligible for full-text screening, which further reduced the number of studies to 61. A flow chart of this search and selection process is presented in Fig. 1, while Appendix 1 provides a full list of the included studies. 32 of these 61 included studies originated in the USA, and all were conducted between 1995 and 2022.

### 3.1 Study characteristics – policy interventions

As Table 1 shows, of the 61 included studies, five of the studied interventions were classified under capacity, 25 under coverage, 20 under managed care, and 11 under payment models. 31 of these 61 interventions had been targeted at children, 17 of which focused on managed care, while 12 of the interventions for adults had focused on coverage. Coverage and managed care interventions were most often created for a specific population groups, while, in contrast, capacity and payment-model policies were often developed for the whole population.

**Table 1:** Interventions categorized by policy category, focus and targeted population group.

Policy category	Intervention focus	Targeted population group					
		Children	Adults	Older people	Pregnant women	All groups	Total
Capacity	Facility investment					1	1
	Financial incentive program					1	1
	Salaried dental teams and delegation of tasks	1					1
	Task delegation					2	2
		**1**				**4**	**5**
Coverage	(Expanded) coverage of dental services	8	11	2	1		22
	Expansion of insurance eligibility to all age groups		1				1
	General anesthesia legislation for dental care	1					1
	Discount on dental insurance copayment			1			1
		**9**	**12**	**3**	**1**		**25**
Managed care	Community-based oral health program	3				1	4
	Educational oral health intervention				2		2
	Pediatric oral health program	9					9
	School-based dental care	5					5
		**17**			**2**	**1**	**20**
Payment models	Capitation	1	1			2	4
	Global budgeting					1	1
	Increased reimbursement for treatment	3					3
	Liberalization of dental price regulations					1	1
	Multi-aspect reform of the public dental care system					1	1
	Pay for performance		1				1
		**4**	**2**			**5**	**11**
Total		**31**	**14**	**3**	**3**	**10**	**61**

*Note*. Numbers in cells refer to quantity of included articles.

### 3.2 Study characteristics – indicators

The studies (n = 61) exhibited 199 indicators (Table 2): 137 focused on accessibility, 21 on affordability, and 41 on oral health status. The indicators ‘dental treatment need’, ‘emergency dental visit’, ‘dental sealant use’, and ‘teeth with decay’ were classified as being associated with more than one goal.

**Table 2:** Frequency table of indicators, classified by intervention goal*.*

Intervention goal	Indicator	Frequency of reported indicator
Accessibility	Age of first dental visit	2
	Dental service	45
	Dental treatment need	1
	Dental visit	26
	Emergency dental visit	2
	Number of dental professionals	3
	Perceived access to dental care	1
	Perceived use of dental care	1
	Preventive dental service	36
	Preventive dental visit	3
	Regular source of dental care	5
	Resignation rate	1
	Restored teeth	2
	Sealant prevalence	2
	Teeth with decay	1
	Fluoride application	1
	Dental sealant use	2
	Curative dental visit	1
	Periodontal treatment	2
		**137**
Affordability	Dental treatment need	1
	Expenditures on oral health	13
	Forgone dental care	2
	Out-of-pocket expenditures on oral health	4
	Inability to afford dental care	1
		**21**
Oral health status	Dental treatment need	5
	Denture needs	1
	Denture wearing	1
	Emergency dental visit	1
	Filled teeth	2
	Missing teeth	2
	Periodontal health	4
	Self-reported oral health	2
	Teeth with decay	11
	DMFT	2
	Dental sealant use	1
	General oral health status	3
	Dental implant use	1
	Mouth pain	1
	Oral health-related quality of life	2
	Plaque index	1
	Curative dental service	1
		**41**
**Total**		**199**

Most accessibility indicators focus on actual use of care, for example, “preventive dental visit” and “periodontal treatment,” with numbers derived from claims data. Other indicators reflected patient characteristics, like “age of first dental visit” or “regular source of dental care.” Subjective indicators were also identified, like “perceived access to dental care” and “perceived use of dental care.” Affordability was measured through indicators directly related to the costs of oral health (here referred to as “expenditures on oral health”) either on a societal level or on a private (out-of-pocket) basis. Likewise, indicators reflecting financial barriers to care, such as “forgone dental care” and “inability to afford dental care,” were also used. For oral health status, the indicators were mostly objective—for example, “dental implant use,” “dental sealant use,” and “decayed, missing, or filled teeth” (DMFT)—but subjective indicators like “self-reported oral health,” “mouth pain,” or “oral health-related quality of life” were also identified.

Because indicators reflecting more than one of the three intervention goals (improving accessibility, affordability, and/or oral health status) were identified in multiple studies, Table 3 provides an overview of the frequency with which each goal was identified within each policy category. For example, of the 25 studies categorized as focusing on coverage policy, 22 included indicators related to accessibility, seven to affordability, and eight to oral health status. This illustrates how one case study could contain indicators associated with different goals.

**Table 3:** Policy categories classified by intervention goals*.*

Policy category	Intervention goals		
	Accessibility (n = 49)	Affordability (n = 9)	Oral health status (n = 21)
Capacity (n = 5)	5	1	0
Coverage (n = 25)	22	7	8
Managed care (n = 20)	13	0	11
Payment models (n = 11)	9	1	2
**Total studies** n = 61			

*Note*. Numbers in cells refer to quantity of included articles.

Table 4 shows the effectiveness of interventions on improving accessibility, affordability or oral health status, categorized by the target groups For studies focused on capacity policy, for example, this table shows that the evaluated interventions were shown to increase the accessibility of dental care (positive reported effect) in five cases: once for children (study 13) and four times for all groups (studies 3, 10, 39, 41). By citing in superscript the reference numbers correlating to the articles listed in Appendix 1, the reader can also see that the same study that reported an increase in accessibility for children (again, study 13) also reported a decrease in pediatric dental care affordability (study 13).

**Table 4:** Reported effects of interventions on accessibility, affordability, or oral health status, classified per targeted population group.

Policy category	Intervention goal	Significance of reported effect	Targeted population group				
			Children	Adults	Older persons	Pregnant women	All groups
**Capacity**	Accessibility	Positive	1^13^				4^3,10,39,41^
		Negative					
		No effect	1^13^				1^10^
	Affordability	Positive					
		Negative	1^13^				
		No effect	1^13^				
	Oral health status	Positive					
		Negative					
		No effect					
**Coverage**	Accessibility	Positive	7^7,34,37,38,52,56,57^	10^1,16,17,24,25,43,44,48,51,53^	1^14^	1^32^	
		Negative		1^48^	1^26^	1^32^	
		No effect	2^30,42^	3^1,51,54^			
	Affordability	Positive	1^56^	3^1,24,25^	1^27^		
		Negative		1^1^	1^27^		
		No effect	1^42^	1^16^			
	Oral health status	Positive	2^37,38^	3^11,16,19^	1^26^		
		Negative		1^11^			
		No effect	2^37,42^	1^17^			
**Managed care**	Accessibility	Positive	9^9,33,36,46,47,49,50,55,61^				1^18^
		Negative	1^29^				
		No effect	3^36,59,61^				
	Affordability	Positive					
		Negative					
		No effect					
	Oral health status	Positive	8^5,8,9,15,28,31,60,61^			2^2,12^	
		Negative					
		No effect	1^59^				
**Payment models**	Accessibility	Positive	3^20,21,40^	1^6^		1^12^	3^6,35,54^
		Negative					3^23,35,54^
		No effect	1^22^				3^23,35,58^
	Affordability	Positive					
		Negative					1^35^
		No effect					
	Oral health status	Positive		2^4,45^			
		Negative					
		No effect					

*Note*. Numbers in cells refer to quantity of included articles. Numbers in superscript refer to reference numbers as reported in Appendix 1.

Studies on managed care for children, and studies on coverage for both children and adults, were identified as having most often reported significant positive effects, specifically: nine studies on managed care (study 9, 33, 36, 46, 47, 49, 50, 55, and 61) and seven studies on coverage (study 7, 34, 37, 38, 52, 56, and 57) reported improvements (significant positive effects) in the accessibility of dental care for children, while 10 coverage studies (study 1, 16, 17, 24, 25, 43, 44, 48, 51, and 53) reported improvements (significant positive effects) in the accessibility of dental care for adults.

## 4 Discussion

This review aimed to scope the available literature, selecting for studies with empirical evaluations of policy interventions that had been developed to improve the accessibility and/or affordability of dental care. All interventions were categorized into one of four policy fields: capacity, coverage, managed care, and payment models.

Three main patterns can be distinguished from our results. The first is that, by redesigning the care delivery process, managed care interventions seem to be effective in improving access to dental care for children. The evaluated managed care interventions included in this review focused on the redesign of dental care delivery and were often implemented within existing community or healthcare settings, for example, at school or as part of general pediatric care. The effects were almost always significantly positive: about one half were shown to positively affect the oral health status of children, while the other half were shown to positively affect the accessibility of pediatric dental care.

The second and third patterns that can be distinguished from our results concern the effectiveness of coverage interventions for improving access to dental care for both (i) children and (ii) adults. Coverage interventions evaluated in the included studies focused either on expansion of the mandated basic health insurance package (e.g., adding services like preventative screening or emergency care) or on expansion of the eligible coverage group (e.g., expanding coverage to other age groups). The results of our review show that these interventions succeeded in improving the accessibility of dental care in almost all cases. A few studies showed significant positive effects on oral health status or affordability as well.

Comparatively, capacity and payment model interventions were less frequently evaluated than managed care and coverage interventions. Capacity interventions—often targeted at the population at large—were in most cases focused on issues of task delegation and shown to have significant positive effects on access to dental care. In studies focused on payment model interventions, analyses were either conducted on different ways of remunerating dentists, for example, through capitation or pay for performance, or on the effects of different price regulations for dental care. For the most part, such financial interventions were found to improve the affordability of dental care.

The patterns described above must not be interpreted as firm conclusions. Firstly, the studies featured in this review employed a broad spectrum of indicators, meaning accessibility, affordability, and oral health status were assessed through various approaches. Secondly, due to the wide range of indicators, our analysis was centered on evaluating the statistical significance of the interventions' reported effects, without examining their actual effectiveness. As a result, it is not feasible to formulate definitive conclusions regarding the overall impact of each policy intervention, and the results of individual interventions should be interpreted with consideration of the specific context of each study.

Extrapolating the three patterns to the context of dental care in the Netherlands, we have reason to expect that implementation of both managed care interventions for children and coverage interventions for adults could potentially produce positive effects. The described effect of coverage interventions improving the accessibility of pediatric dental care will not be considered in this regard, as dental care in the Netherlands is already covered by basic health insurance for all children up to age 18.

In The Netherlands, pediatric managed care interventions are often only implemented locally, and national policies are lacking [24]. This contrasts with many other countries that already have implemented managed care interventions for children [18]. That there is still room for improvement is shown by the fact that, despite full coverage for children, not all minors in the Netherlands visit an oral care provider [9]. Given their proven effectiveness, further expansion of pediatric managed care interventions within the Netherlands may be beneficial. One type of managed care intervention frequently evaluated by the studies included in our review involves the provision of school-based dental care. Although no national policies of this kind are currently in effect within the Netherlands, political attention is given to the subject. In fact, the Dutch House of Representatives recently issued a statement advising further research into which interventions may contribute to the dental visiting behaviors of children, noting that the implementation of school-based dental care [25] deserves special attention. Another type of managed care intervention currently under research in the Netherlands is the community-based intervention, for which a project entitled GigaGaaf!—aimed at improving the oral health status of children—is both implementing and investigating cooperation between well-child clinics and oral health professionals [26]. Implementation of such managed care interventions for children seem necessary given that full coverage of pediatric dental care alone appears unable to guarantee widespread sufficient use of care.

Children from families with a low socioeconomic status are particularly likely to suffer from limited access to oral health care and unfavorable oral health conditions [11,25]. In the most recent Dutch coalition agreement, persisting socioeconomic health differences are specifically mentioned as being undesirable, and the tackling of preventable health differences is a priority [27]. Although the agreement does not explicitly mention dental care, the implementation of managed care interventions aimed at decreasing children's socioeconomic oral health differences would be in accordance with this statement. Considering these differences in health between socioeconomic groups and from an equitable viewpoint, a focus on implementing such interventions in areas with lower socioeconomic rates may prove most effective.

Due to the ongoing socioeconomic disparities in health [28], the potential introduction of public dental insurance for adults has become a subject of political discussion in the Netherlands, particularly regarding the specific treatments that should be included. At this moment, access to dental care for adults is a personal financial matter, as public dental insurance ceases at age 18. Adults can purchase private, supplementary insurance; however, this often does not reimburse enough to cover the comprehensive cost of treatments [29], and further opportunities for financial support are limited. In Europe, this makes the Netherlands rather unique: only Spain, Italy, and Portugal are also almost completely reliant on private funds for the coverage of dental care as well. In most other countries, routine or emergency dental treatments are often publicly covered [30,31].

In line with both our results, and the funding and organization of dental care in other countries, there is reason to believe that the implementation of public insurance for dental examinations, routine treatments like fillings, and some emergency services, such as tooth extractions [30,31], would improve oral health outcomes. A complicating factor in this debate concerns the added cost of covering dental care for adults within the basic health insurance package, as this will likely lead to an increased premium and therefore not automatically resolve the financial barriers to healthcare.

Should there be rises in managed care interventions for children and/or coverage of dental care for adults, this could potentially lead to *spillover effects*—i.e., when the implementation of an intervention for one group leads to effects for another [32,33]. For example, an increase in school-based dental care could raise awareness among parents about the importance of dental health. Vice versa, extending the coverage of dental care for adults, which would likely increase adult dental visit rates, may positively affect the dental visit rate of children as well.

Although our review does not include many studies of capacity interventions, capacity issues in the Dutch dental sector are urgent and may benefit from insights gleaned through the evaluated capacity policies. Of particular relevance may be one currently ongoing nationwide task redistribution program—clearly in line with the capacity policies included in this review—which is expected to lead to a 1.5% shift from dentists to dental hygienists in the performance of certain activities. However, the need to educate dentists on how to better respond to problems of accessibility is expected to remain relevant [15]. According to our review, policy measures could focus on developing incentives to attract more oral care providers to specific regions in which they are scarce. Another intervention focus that could improve capacity problems is teledentistry. While teledentistry interventions were excluded from this review due to selection criteria, evidence suggests that they may help with both a lack of professionals and cost management, for example, by improving the accessibility of diagnostic treatments and referrals [34].

Our scoping review has some limitations. Firstly, we did not do a supplementary search in Google Scholar, due to reasons of feasibility. Doing this is recommended, and it may have improved our search results. Another limitation is that, in our selection of studies that included a quantitative evaluation, we excluded those that made use of machine learning or computational modelling. These methods are fairly new but should be included in future (scoping) reviews. Thirdly, we also excluded studies of interventions aimed at reducing socioeconomic health differences in dental care, choosing instead to focus on interventions for the general public. Although this was justifiable, as the specific goal of our review was not to scope interventions that may decrease socioeconomic health differences, their inclusion may have been relevant given the current socioeconomic health differences within the Netherlands. A last limitation pertains to the data extraction process. Because of (i) the extreme variation in indicators that were used in the studies, (ii) the many ways in which indicators were reported, and (iii) the lack of standardization, we looked only at the level of significance associated with each indicator and not at the specific effect sizes. This makes it hard to draw firm conclusions about the precise effect of the studied interventions.

Further research could focus on the specific policy interventions that, according to our review, may be effective in improving access to dental care and the oral health status of residents in the Netherlands. Thanks to the broad scope of our review, future researchers may benefit from our insights into (the existence of) studies within a certain policy category. However, to assess the methodological quality of the included studies and the effect sizes of their interventions, a systematic review of the interventions included in each specific policy category is needed. In addition, we believe that intensified use of quality indicators in dental care research in general would improve the evaluation and comparison of interventions. Possible spillover effects of policy implementations—which, given their complexity and relation to specific local organization of dental care, may be particularly well suited to computational modelling—also deserve further research.

## 5 Conclusion

Based on this scoping review and given the characteristics of the Dutch dental care system, evidence suggests that implementing managed care interventions for children and expanding the basic health insurance package to include dental coverage for adults may improve access to dental care and oral health status in the Netherlands. Further investigation is needed before solid practical implications can be made. Due to possible spillover effects, it is recommended to investigate a combination of both managed care interventions for children and expanded coverage for adults. Moreover, further research is necessary to determine whether such interventions need only be implemented for population groups with a low socioeconomic status.

## Data availability statement

No data associated with this study has been deposited into a publicly available repository.

## CRediT authorship contribution statement

**E.H. Wolf:** Writing – review & editing, Writing – original draft, Conceptualization, Data curation, Formal analysis, Investigation, Methodology, Visualization. **K.A. Ziesemer:** Methodology, Data curation. **E.J.E. Van der Hijden:** Writing – review & editing, Writing – original draft, Supervision, Formal analysis, Conceptualization.

## Declaration of competing interest

The authors declare that they have no known competing financial interests or personal relationships that could have appeared to influence the work reported in this paper.

## Figures and Tables

**Table:** 

#	Author	Year	Country	Intervention focus	Policy category	Target group	Intervention goal	Indicator category	Indicator	Intended effect direction	S/NS	Significant effect
1 [35]	Abdus e.a.	2019	United States	(expanded) coverage of dental services	coverage	adults	accessibility	dental service	received restorative services	increase	S	positive
									received prosthetic services	increase	S	positive
									received oral surgery services	increase	NS	no effect
									received other dental services	increase	S	positive
								dental visit	dental visit in the past year	increase	S	positive
									number of dental visits per year	increase	S	positive
								preventive dental service	received diagnostic services	increase	S	positive
									received preventive services	increase	S	positive
							affordability	expenditures on oral health	total expenditures on oral health	decrease	S	negative
								out-of-pocket expenditures on oral health	out-of-pocket expenditures on oral health	decrease	S	positive
2 [36]	Adams e.a.	2017	United States	oral health education	managed care	pregnant women	oral health status	periodontal health	sites with bleeding upon probing	decrease	S	positive
									sites with pocket depths ≥4 mm	decrease	S	positive
								plaque index	mean plaque index	decrease	S	positive
3 [37]	Akinlotan e.a.	2022	United States	task delegation	capacity	all	accessibility	emergency dental visit	emergency dental visits	decrease	S	positive
4 [38]	Andås e.a.	2021	Sweden	capitation	payment models	adults	oral health status	teeth with decay	teeth with dental caries	decrease	S	positive
5 [39]	Azevedo e.a.	2015	Brazil	community-based oral health program	managed care	children	oral health status	teeth with decay	teeth with dental caries	decrease	S	positive
6 [40]	Best e.a.	2005	United Kingdom	capitation	payment model	all	accessibility	regular source of dental care	registered children	increase	S	positive
									registered adults	increase	S	positive
7 [41]	Bhagavatula e.a.	2017	United States	(expanded) coverage of dental services	coverage	children	accessibility	dental service	pediatric simple restorative dental visit	decrease	S	positive
									pediatric complex restorative dental visit	decrease	S	positive
									pediatric endodontic dental visit	decrease	S	positive
									pediatric extraction dental visit	decrease	S	positive
								preventive dental service	pediatric diagnostic dental visit	increase	S	positive
									pediatric preventive dental visit	increase	S	positive
8 [42]	Burgette e.a.	2017	United States	pediatric oral health program	managed care	children	oral health status	oral health-related quality of life	negative reported oral health related to quality of life	decrease	S	positive
9 [43]	Carpino e.a.	2016	United States	school-based dental care	managed care	children	accessibility	dental treatment need	treatment urgency (no referral necessary)	decrease	S	positive
								restored teeth	change in number of restored teeth	increase	S	positive
							oral health status	teeth with decay	change in number of teeth with decay	decrease	S	positive
10 [44]	Chi e.a.	2018	United States	task delegation	capacity	all	accessibility	dental service	pediatric extraction dental visit	decrease	S	positive
									pediatric extraction dental visit	decrease	S	positive
									pediatric general anesthesia	decrease	NS	no effect
									pediatric general anesthesia	decrease	S	positive
									adult extraction	decrease	S	positive
									adult extraction	decrease	S	positive
								preventive dental service	child preventive dental care	increase	S	positive
									pediatric preventive dental care	increase	S	positive
									adult preventive dental care	increase	S	positive
									adult preventive dental care	increase	S	positive
11 [45]	Choi e.a.	2020	Korea	(expanded) coverage of dental services	coverage	adults	oral health status	dental treatment need	number of people in need of dental scaling	decrease	S	positive
								periodontal health	prevalence of healthy periodontal tissue	increase	S	positive
									prevalence of periodontal disease	decrease	S	negative
12 [46]	Cibulka e.a.	2009	United States	oral health education	managed care	pregnant women	accessibility	dental visit	dental visit	increase	S	Positive
							oral health status	self-reported oral health	self-reported oral health	increase	S	positive
13 [47]	Conrad e.a.	2021	United States	salaried dental teams and delegation of tasks	capacity	children	accessibility	dental service	any dental service	increase	NS	positive
									any restorative service	increase	NS	no effect
									any extraction service	decrease	NS	no effect
								preventive dental service	any preventive service	increase	NS	no effect
									any diagnostic service	increase	NS	no effect
									application of fluoride varnish	increase	NS	no effect
									dental sealants	increase	S	positive
									caries arrest by Silver Diamine Fluoride	increase	NS	no effect
							affordability	expenditures on oral health	cost of dental service (all types)	decrease	S	negative
									cost of preventive dental service (all types)	decrease	NS	no effect
									cost of diagnostic dental service (all types)	decrease	NS	no effect
									cost of fluoride varnish application	decrease	NS	no effect
									cost of dental sealants	decrease	S	negative
									cost of caries arrest by Silver Diamine Fluoride	decrease	NS	no effect
									cost of restorative dental service (all types)	decrease	NS	no effect
									cost of tooth extraction service (all types)	decrease	NS	no effect
14 [48]	Cooray e.a.	2020	Japan	discount on dental health insurance copayment	coverage	older people	accessibility	dental service	treatment visits in past 12 months	increase	S	positive
								preventive dental service	check-up visits in past 12 months	increase	S	positive
15 [49]	Dabiri e.a.	2016	El Salvador	community-based oral health program	managed care	children	oral health status	mouth pain	mouth pain	decrease	S	positive
								teeth with decay	caries experience	decrease	S	positive
16 [50]	Decker e.a.	2015	United States	(expanded) coverage of dental services	coverage	adults	accessibility	dental visit	yearly dental visit	increase	S	positive
							affordability	forgone dental care	No dental visit due to cost	decrease	S	positive
							oral health status	missing teeth	broken or missing teeth	decrease	S	positive
								teeth with decay	untreated caries	decrease	S	positive
17 [51]	Elani e.a.	2021	United States	(expanded) coverage of dental services	coverage	adults	accessibility	dental visit	dental visit in the past year	increase	S	positive
							affordability	inability to afford dental care	inability to afford dental care	decrease	NS	no effect
							oral health status	filled teeth	filled teeth	increase	NS	no effect
								oral health status	fair or poor reported oral health	decrease	NS	no effect
								teeth with decay	untreated teeth with decay	decrease	NS	no effect
18 [52]	Frazão e.a.	2008	Brazil	community-based oral health program	managed care	all	accessibility	perceived access to dental care	perceived family access to public dental services	increase	S	positive
								perceived use of dental care	perceived family use of public dental services	increase	S	positive
19 [53]	Gnanamanickam e.a.	2017	Australia	(expanded) coverage of dental services	coverage	adults	oral health status	filled teeth	filled teeth	decrease	S	positive
								missing teeth	missing teeth	decrease	S	positive
								teeth with decay	decayed teeth	decrease	S	positive
20 [54]	Gnich e.a.	2018	Scotland	increased treatment reimbursement	payment models	children	accessibility	preventive dental service	rate of fluoride varnish application on teeth of children 2–5 years old	increase	S	positive
21 [55]	Grembowski e.a.	2006	United States	capitation	payment models	children	accessibility	dental sealant use	number of sealants applied per child	increase	NS	positive
								fluoride application	number of fluoride applications per child	increase	NS	positive
								restored teeth	number of restorations per child	decrease	S	positive
22 [56]	Griffin e.a.	2007	United States	increased treatment reimbursement	payment models	children	accessibility	sealant prevalence	sealant prevalence	increase	S	no effect
									sealant prevalence	increase	S	no effect
23 [57]	Hill e.a.	2017	United Kingdom	capitation	payment models	all	accessibility	dental service	extractions per 100 patients seen	decrease	S	negative
									fillings per 100 patients seen	increase	S	negative
								dental visit	patients seen per 100 registrations	increase	S	negative
								preventive dental service	examinations per 100 patients seen	increase	S	negative
									scale and polish per 100 patients seen	increase	S	negative
									fluoride varnish application per 100 patients seen	increase	NS	no effect
24 [58]	Kempe e.a.	2020	United States	(expanded) coverage of dental services	coverage	adults	accessibility	emergency dental visit	emergency dental visits	decrease	S	positive
							affordability	out-of-pocket expenditures on oral health	self-pay for emergency dental visits	decrease	S	positive
25 [59]	Kim e.a	2019	Korea	(expanded) coverage of dental services	coverage	adults	accessibility	preventive dental service	dental scaling use	increase	S	positive
26 [60]	Kim e.a.	2021	Korea	(expanded) coverage of dental services	coverage	elderly	accessibility	preventive dental visit	oral examination	increase	S	negative
							oral health status	dental implant use	dental implant use	increase	S	positive
								dental treatment need	dental treatment need	increase	S	positive
								denture needs	denture needs	decrease	S	positive
								denture wearing	denture wearing	increase	S	positive
27 [61]	Kim e.a.	2021	Korea	(expanded) coverage of dental services	coverage	elderly	affordability	out-of-pocket expenditures on oral health	dental care expenditures on denture services	decrease	S	positive
									dental care expenditures on dental implants	decrease	S	negative
28 [62]	Kranz e.a.	2015	United States	pediatric oral health program	managed care	children	oral health status	dmft	oral health status (dmft)	decrease	S	positive
29 [63]	Kranz e.a.	2015	United States	pediatric oral health program	managed care	children	accessibility	age of first dental visit	dental visit before age 3	decrease	S	negative
30 [64]	Kranz e.a.	2018	United States	(expanded) coverage of dental services	coverage	children	accessibility	dental visit	pediatric dental visit in the past year	increase	NS	no effect
31 [65]	Kranz e.a.	2019	United States	pediatric oral health program	managed care	children	oral health status	self-reported oral health	oral health status (report of “very good” or “excellent” teeth by parents)	increase	S	positive
32 [66]	Lee e.a.	2021	United States	(expanded) coverage of dental services	coverage	pregnant women	accessibility	dental service	dental cleaning	increase	S	positive
									dental visit	decrease	S	negative
33 [67]	Lewis e.a.	2009	United States	pediatric oral health program	managed care	children	accessibility	preventive dental service	preventive dental visits	increase	S	positive
34 [68]	Liao e.a.	2008	United States	(expanded) coverage of dental services	coverage	children	accessibility	preventive dental visit	likelihood of receiving preventive dental care	increase	S	positive
35 [69]	Lin e.a.	2012	Taiwan	global budgeting	payment models	all	accessibility	dental service	root canal procedures in hospitals	increase	S	positive
									root canal procedures in outpatient dental clinics	increase	NS	no effect
									ionomer restorations in hospitals	increase	S	positive
									ionomer restorations in outpatient dental clinics	increase	S	positive
									tooth extractions in hospitals	decrease	S	negative
									tooth extractions in outpatient dental clinics	decrease	S	negative
									amalgam restorations in hospitals	decrease	NS	no effect
									amalgam restorations in outpatient dental clinics	decrease	S	positive
								dental visit	number of visits per patient	increase	NS	no effect
								preventive dental service	tooth scaling in hospitals	increase	S	positive
									tooth scaling in outpatient dental clinics	increase	S	positive
							affordability	expenditures on oral health	reimbursement per dental visit	decrease	S	negative
									reimbursement per patient	decrease	S	negative
									reimbursement per dentist	decrease	S	negative
36 [70]	Martinelli e.a.	2020	Brazil	community-based oral health program	managed care	children	accessibility	dental service	use of public dental services	increase	S	positive
									use of preventive dental services	increase	NS	no effect
37 [71]	McBroome e.a.	2003	United States	(expanded) coverage of dental services	coverage	children	accessibility	regular source of dental care	regular source of dental care	increase	S	positive
							oral health status	dental treatment need	need for dental care	decrease	NS	no effect
									unmet need for dental care	decrease	S	positive
38 [72]	Mofidi e.a.	2002	United States	(expanded) coverage of dental services	coverage	children	accessibility	dental visit	number of pediatric recipients of dental care in the past year	increase	S	positive
								regular source of dental care	children with a regular source of dental care	increase	S	positive
							oral health status	dental treatment need	children with dental needs	decrease	S	positive
39 [73]	Nguyen e.a.	2002	United States	facility investment	capacity	all	accessibility	dental visit	dental visit	increase	S	positive
								number of dental professionals	number of dental professionals (dentists)	increase	S	positive
									number of dental professionals (dental hygienists)	increase	S	positive
									number of dental professionals (dental assistants)	increase	S	positive
								regular source of dental care	registered dental patients	increase	S	positive
40 [74]	Nietert e.a.	2005	United States	increased treatment reimbursement	payment models	children	accessibility	dental service	pediatric restorative dental services (0–2 years old)	increase	S	positive
									pediatric surgical dental services (0–2 years old)	increase	S	positive
									pediatric restorative dental services (3–21 years old)	increase	S	positive
									pediatric surgical dental services (3–21 years old)	increase	S	positive
								preventive dental service	pediatric diagnostic dental services (0–2 years old)	increase	S	positive
									pediatric preventive dental services (0–2 years old)	increase	S	positive
									pediatric diagnostic dental services (3–21 years old)	increase	S	positive
									pediatric preventive dental services (3–21 years old)	increase	S	positive
41 [75]	Noochpoung e.a.	2022	Thailand	financial incentive program	capacity	all	accessibility	resignation rate	resignation rate per 100 dentists	decrease	S	positive
42 [76]	Ono e.a.	2021	Japan	(expanded) coverage of dental services	coverage	children	accessibility	age of first dental visit	first dental visit	increase	NS	no effect
								dental visit	total dental visits	increase	NS	no effect
							affordability	expenditures on oral health	dental costs	decrease	NS	no effect
							oral health status	emergency dental visit	visits for severe dental conditions	decrease	NS	no effect
43 [77]	Osterberg e.a.	1995	Sweden	(expanded) coverage of dental services	coverage	adults	accessibility	dental service	dental service utilization (men)	increase	S	positive
									dental service utilization (women)	increase	S	positive
44 [78]	Park e.a.	2018	Korea	(expanded) coverage of dental services	coverage	adults	accessibility	periodontal treatment	dental service utilization for the treatment of gingivitis (expressed in total treatment costs)	increase	S	positive
45 [79]	Pavitt e.a.	2016	United Kingdom	pay for performance	payment models	adults	oral health status	curative dental service	extractions and fillings	decrease	S	positive
								oral health-related quality of life	oral health-related quality of life	increase	S	positive
46 [80]	Pinilla e.a.	2006	Spain	pediatric oral health program	managed care	children	accessibility	dental visit	dental visits in the past year	increase	S	positive
47 [81]	Quinonez e.a.	2018	United States	pediatric oral health program	managed care	child	accessibility	dental visit	mean number of monthly clinic visits	increase	S	positive
48 [82]	Raittio e.a.	2014	Finland	expansion of insurance eligibility to all age groups	coverage	adults	accessibility	dental service	number of provided oral health care services (all types)	increase	S	negative
								dental visit	% use of provided oral health care services (all types)	increase	S	positive
									regular dental visiting habits	increase	S	positive
49 [83]	Sanjeevan e.a.	2019	India	school-based dental care	managed care	children	accessibility	dental visit	dental attendance	increase	S	positive
50 [84]	Seirawan e.a.	2020	United States	pediatric oral health program	managed care	children	accessibility	dental visit	scheduled dental appointment	increase	S	positive
51 [85]	Shane e.a.	2017	United States	(expanded) coverage of dental services	coverage	adults	accessibility	curative dental visit	receiving dental treatments	increase	S	positive
								periodontal treatment	dental service utilization for the treatment of advanced periodontitis (expressed in total treatment costs)	increase	S	positive
								preventive dental visit	use of preventive dental services	increase	NS	no effect
52 [86]	Sohn e.a.	2019	Korea	(expanded) coverage of dental services	coverage	children	accessibility	preventive dental service	pediatric dental sealant use	increase	S	positive
								teeth with decay	pediatric prevalence of untreated caries	decrease	S	positive
53 [87]	Suominen e.a.	2017	Finland	(expanded) coverage of dental services	coverage	adults	accessibility	dental visit	use of any oral health care within the previous year	increase	S	positive
									visit to ‘Public Dental Service’ dentist within the previous year	increase	S	positive
									visit to private dentist within the previous year	increase	NS	no effect
54 [88]	Trescher e.a.	2019	the Netherlands	liberalization of dental cost regulations	payment models	all	accessibility	dental service	adult restorative dental service	increase	S	negative
									adult extraction dental service	increase	S	positive
									adult radiograph dental service	increase	S	negative
									pediatric restorative dental service	increase	S	negative
									pediatric extraction dental service	increase	S	negative
									pediatric radiograph dental service	increase	S	positive
								preventive dental service	adult preventive dental service	increase	S	negative
									adult scaling and polishing	increase	S	positive
									adult preventive exam and oral hygiene advice	increase	S	negative
									pediatric preventive dental service	increase	S	negative
									pediatric preventive exam and oral hygiene advice	increase	S	negative
									pediatric scaling and polishing	increase	S	negative
									pediatric sealants	increase	S	negative
55 [89]	Trudnak e.a.	2018	United States	school-based dental care	managed care	children	accessibility	preventive dental service	dental visit for teeth cleaning within the past year	increase	S	positive
									receiving overall preventive services	increase	S	positive
56 [90]	Wang e.a.	2007	United States	(expanded) coverage of dental services	coverage	children	accessibility	dental visit	most recent dental visit within the past 6 months	increase	S	positive
					coverage				most recent dental visit within the past 12 months	increase	S	positive
							affordability	dental treatment need	unmet dental need	decrease	S	positive
								forgone dental care	unmet dental need due to cost	decrease	S	positive
57 [91]	White e.a.	2008	United States	general anesthesia legislation for dental care	coverage	children	accessibility	dental visit	pediatric dental visits	increase	S	positive
58 [92]	Whittaker e.a.	2012	United Kingdom	Multi-aspect reform of the public dental care system	payment models	all	accessibility	dental visit	NHS dental care use	increase	NS	no effect
59 [93]	Williams e.a.	2017	United States	school-based dental care	managed care	children	accessibility	dental sealant use	sealant prevalence	increase	NS	no effect
							oral health status	teeth with decay	active caries	decrease	NS	no effect
60 [94]	Wolff e.a.	2016	Grenada	school-based dental care	managed care	children	quality	teeth with decay	average number of decayed teeth surfaces	decrease	S	positive
									average number of demineralized teeth surfaces	decrease	S	positive
61 [95]	Yuan e.a.	2018	China	pediatric oral health program	managed care	children	accessibility	dental visit	most recent dental visit within the past 6 months	increase	S	positive
									most recent dental visit more than 12 months ago	decrease	S	negative
							oral health status	dental sealant use	present sealants	increase	S	positive
								DMFT	oral health status (% of children with DMFT = 0)	increase	S	positive
								oral health status	purpose of most recent dental visit (diagnostic)	increase	S	positive
									purpose of most recent dental visit (curative)	decrease	S	positive
								teeth with decay	caries experience (% of children with decayed teeth)	decrease	S	positive

∗ Reference numbers are also listed in the reference section found in Appendix 2.
